# Effect of Anterior Chamber Instability during Phacoemulsification Combined with Intraocular Lens Implantation

**DOI:** 10.1155/2022/2848565

**Published:** 2022-02-23

**Authors:** Wenjing Geng, Wenli Lin, Pei Song, Min Zhang, Jian Wu, Shu Su, Yurong Yuan, Min Ji, Huaijin Guan

**Affiliations:** Eye Institute, Affiliated Hospital of Nantong University, Nantong 226000, Jiangsu, China

## Abstract

**Purpose:**

To determine the incidence of anterior chamber (AC) instability during phacoemulsification (phaco) combined with intraocular lens implantation and investigate its effect on intraocular tissues.

**Methods:**

Among the 248 enrolled eyes, 121 and 127 eyes were categorized into the irrigation and nonirrigation groups, respectively, depending on the use of a self-made anterior chamber maintainer (ACM) during phaco. AC stability was evaluated using operating microscopy and intraoperative optical coherence tomography (iOCT). Slit-lamp examination of AC flare and cells was performed 1 day postoperatively. Corrected distance visual acuity (CDVA), intraocular pressure (IOP), endothelial cell density (ECD), central corneal thickness (CCT), posterior vitreous detachment (PVD), and central foveal thickness (CFT) were evaluated preoperatively and at 1 week, 1 month, and 3 months postoperatively.

**Results:**

There was good consistency in AC stability evaluation between operating microscopy and iOCT. The incidence of AC instability was significantly different between groups after the phaco and irrigation/aspiration tips were withdrawn from the AC (*P* < 0.001). At 1 day postoperatively, after excluding eyes in which the AC could not be visualized, AC cell grades were significantly lower in the irrigation group (*P* = 0.014). There was no significant difference in CDVA, IOP, ECD, and CCT between groups during the 3-month follow-up (*P* > 0.05). At 1 month and 3 months postoperatively, PVD occurred in 8 (16.3%) and 14 (28.6%) eyes and in 22 (40.7%) and 37 (68.5%) eyes in the irrigation and nonirrigation groups, respectively (*P* = 0.006 and *P* < 0.001). CFT was significantly higher in the nonirrigation group at 1 and 3 months postoperatively (*P* = 0.018 and *P* = 0.010).

**Conclusions:**

Both operating microscopy and iOCT are efficient for AC stability evaluation. When the phaco and I/A tips were withdrawn from the AC, there was frequent instability. Intraoperative AC instability can increase surgery-induced inflammation and lead to postoperative complications such as PVD, retinal detachment, and macular edema. The self-made ACM can effectively reduce the incidence of intraoperative AC instability and these complications.

## 1. Introduction

Currently, phacoemulsification (phaco) combined with intraocular lens (IOL) implantation is the gold standard for cataract treatment [1]. However, several factors affect the success of surgery, leading to intraoperative and postoperative complications. Among them, anterior chamber (AC) stability during phaco is a critical factor [2].

The balance between the inflow and outflow of irrigation fluid primarily influences AC stability. Modern phaco machines can maintain a relatively stable AC when the phaco tip and the irrigation/aspiration (I/A) tip are inserted into the eyes [3]. However, the AC immediately becomes shallow or even collapses once the surgical instruments are withdrawn from it.

Here, we have presented a self-made anterior chamber maintainer (ACM) designed to maintain AC stability. Although maintaining AC stability using an ACM is not a novel concept, previous studies [4, 5] have evaluated the effectiveness of the ACM by subjective assessment of the posterior capsule position. Several studies [6, 7] have evaluated the effect of using ACM on the corneal endothelium; however, to our knowledge, no study has evaluated the effect of AC stability on the posterior segment.

Therefore, we aimed to determine the incidence of AC instability during phaco combined with IOL implantation using two devices and investigate its effect on intraocular tissues such as the cornea, vitreous, and retina.

## 2. Patients and Methods

### 2.1. Patients

This prospective comparative study comprised patients with age-related cataract (ARC) who received phaco at the Department of Ophthalmology, Affiliated Hospital of Nantong University, from May 2020 to May 2021. This study was conducted according to the Declaration of Helsinki and approved by the Institutional Review Committee of the Affiliated Hospital of Nantong University. All patients provided written informed consent before surgery. The inclusion criteria were lens nuclear opalescence grade 2-3 on the Lens Opacities Classification System (LOCS) III classification and attending follow-up visits for at least 3 months postoperatively.

The exclusion criteria were as follows: poor pharmacological mydriasis (<7 mm), endothelial cell density (ECD) <1500 cells/mm^2^, media opacities that did not allow OCT images to be obtained with an image quality >6/10, and previous ocular surgery or trauma. Patients were categorized into the irrigation and nonirrigation groups depending on the use of the self-made ACM during surgery.

### 2.2. Preoperative Evaluation

Patients underwent a series of ophthalmological examinations, including slit-lamp and dilated fundus examinations. Central corneal thickness (CCT) and ECD were measured using a corneal specular operating microscope (EM-3000; Tomey Corp.). Biometric parameters including axial length (AL) and anterior chamber depth (ACD) were measured using a noncontact optical biometry device (LS900; Haag-Streit AG). The degree of posterior vitreous detachment (PVD) was evaluated using OCT (Cirrus HD-5000; Carl Zeiss Meditec AG) and B-scan ultrasonography (US) (Aviso; Quantel Medical). Macular thickness was objectively measured using OCT.

### 2.3. Making the ACM

**Figure 1:**
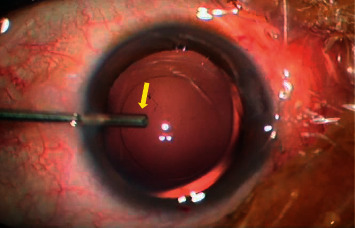
The self-made anterior chamber maintainer irrigation tip (yellow arrows).

The self-made ACM consists of three parts—a self-made AC irrigation tip, a single-use transfusion set, and an irrigation bottle. We bent the first one-third part of the 22-gauge syringe needle (outer needle diameter: 0.7 mm) by 30°, ground the front end of the needle with a dental handpiece (ULTIMATE 500, NSK Ltd.), and polished it with tungsten carbide burs (MANI Inc.) to make the needle rounded and smooth (Figure 1).

### 2.4. Posterior Vitreous Detachment Grading

**Figure 2:**
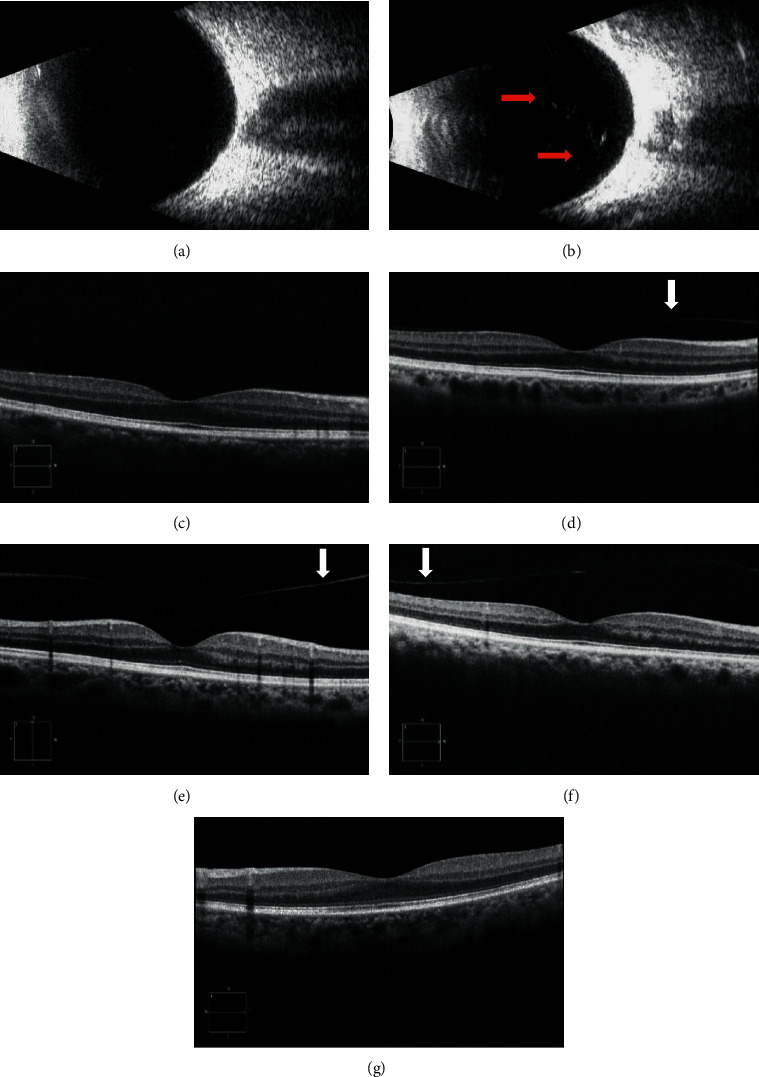
Posterior vitreous detachment (PVD) grading based on B-scan ultrasonography (US) and optical coherence tomography (OCT). (a) No PVD, B-scan US showing the absence of a hyperechoic membrane over the retinal pigment epithelium (RPE) layer. (b) Complete PVD, presence of a hyperechoic membrane (red arrows) in addition to the posterior RPE layer and completely detached from the RPE layer. (c) Stage 0, no PVD. (d) Stage 1, focal perifoveal PVD involving one to three quadrants. (e) Stage 2, perifoveal PVD across all quadrants, with persistent attachment to the fovea. (f) Stage 3, detachment of the posterior vitreous face from the fovea, with persistent attachment to the optic disc. (g) Stage 4, complete detachment of the posterior vitreous cortex (PVC) from the retina and optic disc, the PVC is not observed. White arrows denote the detached PVC.

B-scan US and OCT images were reviewed and evaluated by a single examiner for PVD staging. All B-scan US images were centered on the optic disc shadow. We classified the PVD status according to the B-scan US as follows: (1) no PVD: absence of a hyperechoic membrane over the retinal pigment epithelium (RPE) layer (Figure 2(a)) and (2) complete PVD: the presence of a hyperechoic membrane in addition to the posterior RPE layer and fully detached from the RPE layer (Figure 2(b)). PVD staging was performed using OCT images according to a previous study [8] as follows: stage 0, no PVD (Figure 2(c)); stage 1, focal perifoveal PVD involving one to three quadrants (Figure 2(d)); stage 2, perifoveal PVD across all quadrants, with persistent attachment to the fovea (Figure 2(e)); stage 3, detachment of the posterior vitreous face from the fovea, with persistent attachment to the optic disc (Figure 2(f)); and stage 4, complete detachment of the posterior vitreous cortex from the retina and optic disc (Figure 2(g)). However, OCT at stage 4 failed to detect any discrete linear signal because the distance from the retina was outside the OCT range. Partial and complete or no PVD were determined based on OCT and B-scan US findings, respectively.

### 2.5. Macular Thickness Measurements

First, we performed a macular cube scan. The average retinal thickness within the foveal 1 mm diameter circle was recorded as the central foveal thickness (CFT). We then used the radial-raster scan protocol centered on the macula to acquire 12 high-resolution images. Macular edema was defined as a >30% increase in CFT from baseline on OCT [9]. To ensure the accuracy of the study, the images with quality <7/10 were eliminated.

### 2.6. Surgical Technique and Interventions

The pupils were dilated with 0.5% tropicamide, and topical anesthesia was administered with 0.5% proparacaine hydrochloride (ALCAINE) before surgery. All surgeries were performed by the same experienced surgeon using a standardized phaco surgical technique (Centurion, Alcon).

In the irrigation group, a transfusion set and an irrigation bottle were hung on the infusion stand, and the infusion set dropper level was 70 cm above the patient's eye level. The transfusion set was filled with irrigation fluid and free of air bubbles. The assistant closed the valve of the transfusion set and connected a self-made AC irrigation tip.

A 2.4 mm clear corneal main incision was made at the steep axis of the cornea under the guidance of the VERION Image Guided System (Alcon) with a keratome. The AC was filled with ophthalmic viscosurgical device (OVD), and continuous curvilinear capsulorhexis (CCC) was followed by hydrodissection and hydrodelineation. After hydrodelineation, a 1.0 mm cornea side incision was made. The second irrigation bottle was opened, and the irrigation tip was inserted through the side incision. Subsequently, a microcoaxial phaco was used to remove the nucleus. When the surgeon withdrew the phaco tip from the AC, the irrigation tip remained in the eye under continuous irrigation. Cortical removal was then performed, while the irrigation tip was continuously perfused. Before IOL implantation, we closed the transfusion set valve while injecting the OVD into the AC. A hydrophobic foldable IOL was implanted. The corneal incisions were hydrated, and the OVDs were removed by I/A.

In the nonirrigation group, the initial steps were similar to those used in the irrigation group. After the cornea side incision was made, the chopper was introduced into the AC from the side incision to assist nuclear chopping during phaco. The surgery was concluded with phaco and IOL implantation, as previously explained. Intraoperative parameters, such as cumulative dissipated energy (CDE), ultrasonic power (USP), ultrasonic total time (UST), and the duration of phaco, were recorded. The duration of phaco was defined from the moment the first quadrant was engaged and aspiration increased to the moment the last quadrant was aspirated.

### 2.7. Intraoperative AC Stability Evaluation

Intraoperative AC stability was evaluated by a single researcher. For intraoperative evaluation, operating microscopy (OPMI Lumera 700, Carl Zeiss Meditec AG) and performed intraoperative OCT (iOCT) (RESCAN700, Carl Zeiss Meditec AG) were used. iOCT images were obtained using cube scans during all surgical steps. We recorded AC changes in the groups at five time points—T1, after making the main incision and before injecting the OVD; T2, at the time of CCC; T3, after the phaco tip was withdrawn from the AC; T4, after aspirating the cortex and withdrawing the I/A tip from the AC; and T5, after aspirating the OVD and withdrawing the I/A tip from the AC.

**Figure 3:**
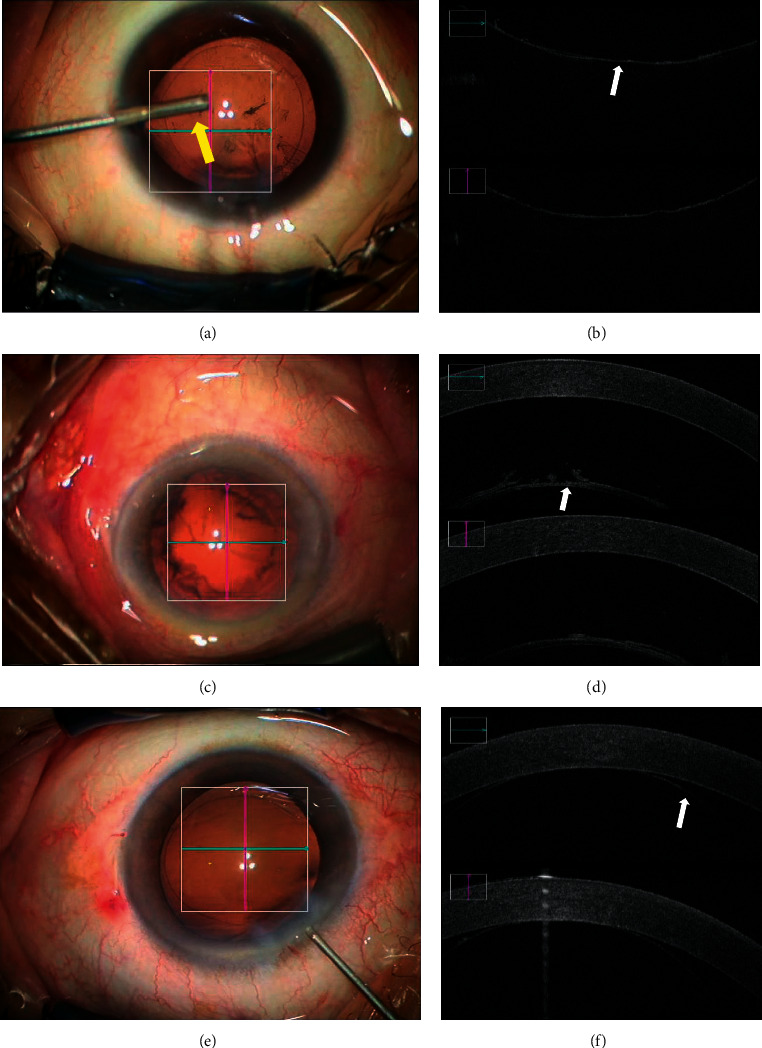
Anterior chamber (AC) stability was evaluated by operating microscopy and intraoperative OCT (iOCT). (a) No forward movement of the posterior capsule (PC) was observed by operating microscopy. (b) A concave arcuate shape of the PC (white arrow) was observed by iOCT. (c) Forward movement of the PC was observed by operating microscopy. (d) Forward movement of the PC (white arrow) was observed by iOCT. (e) The disappearance of the AC was observed by operating microscopy. (f) The PC (white arrow) contact with the corneal endothelium was observed by iOCT. The yellow arrow denotes the self-made AC irrigation tip.

At T1 and T2, we observed the position of the iris-lens diaphragm using operating microscopy and iOCT, respectively, to determine the changes in the AC. We defined the iris-lens diaphragm in situ as AC stability and forward displacement as AC instability. After nuclear emulsification (T3, T4, and T5), we observed the position of the posterior capsule (PC). On operating microscopy, no forward movement of the PC was defined as AC stability (Figure 3(a)) and forward movement of the PC or even disappearance of the AC was defined as AC instability (Figures 3(c) and 3(e)). On iOCT, a concave arcuate shape of the PC was defined as AC stability (Figure 3(b)) and anterior movement of the PC or contact with the corneal endothelium was defined as AC instability (Figures 3(d) and 3(f)).

### 2.8. Postoperative Evaluation

AC cells and flare were graded at 1 day postoperatively according to the Standardized Uveitis Nomenclature [10] by the same evaluator using a 1.0 × 1.0 mm slit beam and graded as follows: cells: 0 = no cells, 0.5 = 1–5 cells, 1 = 6–15 cells, 2 = 16–25 cells, 3 = 26–50 cells, 4 ≥ 50 cells; flare: 0 = none, 1 = faint, 2 = moderate, iris and lens detail clear, 3 = marked, iris and lens detail hazy, 4 = intense, fibrin or plastic aqueous. CDVA, IOP, CCT, ECD, degree of PVD, and CFT were assessed at 1 day, 1 month, and 3 months postoperatively.

### 2.9. Statistical Analysis

Statistical analysis was performed using IBM SPSS Statistics for Windows (version 26.0, IBM Corp.). Data are presented as mean ± standard deviation. Visual acuity data were converted to the logarithm of the minimal angle of resolution to calculate the mean. For analysis of continuous variables, Student's *t-*test and the nonparametric test were used for normally distributed and nonparametric variables, respectively.

The chi-square test was used to analyze categorical variables (LOCS cataract and PVD grade). The Mann–Whitney *U* test was used to compare AC flare and cell values between groups.

The level of agreement between operating microscopy and iOCT was analyzed using the Kappa test. Kappa values of 1 represent perfect agreement, and values close to 1 indicate high levels of agreement. Kappa values ≥0.75, 0.4–0.75, and <0.4 indicated high, moderate, and low agreement, respectively.

## 3. Results

We enrolled 242 patients (280 eyes), 140 eyes each in the irrigation and nonirrigation groups. In total, 121 (86.4%) and 127 (90.7%) eyes completed the 3-month follow-up in the irrigation and nonirrigation groups. Preoperatively, there was no significant difference between groups in terms of age, sex, IOP, nuclear grade, AL, and ACD (Table 1).

All surgical procedures were uneventful. The CDE, USP, and UST were 3.88 ± 2.95, 25.94 ± 17.02, and 20.06 ± 15.79 s, respectively, in the irrigation group and 3.57 ± 2.94, 23.10 ± 19.98, and 23.01 ± 16.83 s, respectively, in the nonirrigation group. No significant differences were noted between groups (*P* > 0.05). The median duration of phaco was 54.5 (34.3, 76) s in the irrigation group and 64 (44, 96) s in the nonirrigation group, with no significant difference between the two median values (*P* = 0.056).

Table 2 shows the incidence of AC instability at five time points during phaco using operating microscopy and iOCT. On operating microscopy, the incidence of AC instability was 6.6% and 73.2% in the irrigation and nonirrigation groups, respectively, at T3 and 10.7% and 89.0% in the irrigation and nonirrigation groups, respectively, at T4. On iOCT, the incidence of AC instability was 7.4% and 77.2% in the irrigation and nonirrigation groups, respectively, at T3 and 14.0% and 92.9% in the irrigation and nonirrigation groups, respectively, at T4. Irrespective of the evaluation technique used, the incidence of AC instability was significantly different between groups at T3 and T4 (*P* < 0.001). According to the results of the iOCT, a stable AC at all five time points was defined as AC stability, whereas an unstable AC at any time point was defined as AC instability. There were 102 (84.3%) and 4 (3.2%) eyes with stable AC at all five time points in the irrigation and nonirrigation groups, respectively (*P* < 0.001) (Table 2).

In the irrigation group, there were moderate and high agreements between operating microscopy and iOCT for AC stability evaluation at T1, T2, and T3 (*κ* = 0.659, 0.740, and 0.684, respectively) and at T4 and T5 (*κ* = 0.772 and 0.839, respectively), respectively (Table 3). In the nonirrigation group, there were moderate and high agreements between the evaluation techniques for AC stability evaluation at T1 and T4 (*κ* = 0.742 and 0.572, respectively) and at T2, T3, and T5 (*κ* = 0.890, 0.768, and 0.779, respectively), respectively (Table 4).

At 1 day postoperatively, slit-lamp examination revealed corneal edema in 14 (11.6%) and 17 (13.4%) eyes in the irrigation and nonirrigation groups, respectively. After excluding eyes in which the AC could not be visualized, AC cell grades were significantly lower in the irrigation group (*P* = 0.014), and no significant differences were observed in flare grades between groups (Table 5).

Preoperative and postoperative CDVA, IOP, ECD, and CCT measurements of the irrigation and nonirrigation groups are presented in Table 6. There was no significant difference in CDVA, IOP, ECD, and CCT between groups during the 3-month follow-up (*P* > 0.05). Compared to baseline, CDVA significantly improved at all follow-up time points in both groups (*P* < 0.001). IOP significantly increased at 1 week postoperatively in both groups, but it decreased at 1 month. In both groups, ECD decreased significantly at 1 week, 1 month, and 3 months postoperatively compared to preoperative values (*P* < 0.001). CCT significantly increased at 1 week (*P* < 0.001) and 1 month (*P* < 0.05) postoperatively, and the values returned to preoperative levels at 3 months postoperatively (*P* > 0.05) (Table 6).

**Figure 4:**
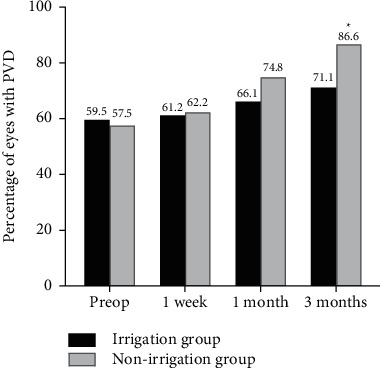
Percentage of eyes with posterior vitreous detachment preoperatively and at 1 week, 1 month, and 3 months postoperatively (^*∗*^*P* = 0.015). PVD: posterior vitreous detachment. ^*∗*^A significant *P* value.

Preoperatively, 49 (40.5%) and 54 (42.5%) eyes in the irrigation and nonirrigation groups, respectively, had no PVD. In the irrigation group, 15 (12.4%), 7 (5.8%), 3 (2.5%), and 47 (38.8%) eyes had stages 1, 2, 3, and 4 PVD, respectively, whereas in the nonirrigation group, 13 (10.2%), 14 (11.0%), 0 (0%), and 46 (36.2%) eyes had stages 1, 2, 3, and 4 PVD, respectively. In two groups of eyes without preoperative PVD, some degree of PVD was observed in 2 (4.1%), 8 (16.3%), and 14 (28.6%) eyes at postoperative 1 week, 1 month, and 3 months, respectively, in the irrigation group and 6 (11.1%), 22 (40.7%), and 37 (68.5%) eyes at postoperative 1 week, 1 month, and 3 months, respectively, in the nonirrigation group (*P* = 0.274, *P* = 0.006, and *P* < 0.001). There was a significantly higher proportion of eyes with PVD at 3 months postoperatively in the nonirrigation group than in the irrigation group (*P* = 0.003) (Figure 4).

**Figure 5:**
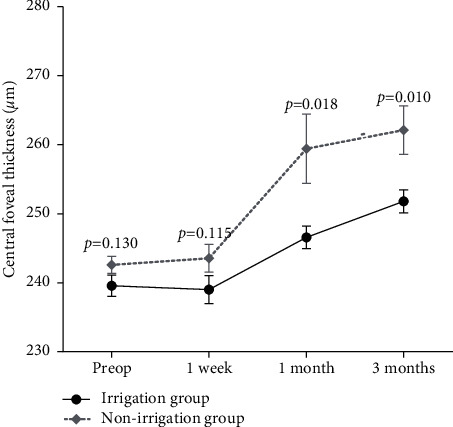
The graph represents the means and the standard errors of the central foveal thickness (*μ*m). The reported *P* values are significant comparisons between the two groups at each time point.

Figure 5 shows the preoperative and postoperative CFT values in the irrigation and nonirrigation groups. CFT was significantly higher in the nonirrigation group at 1 and 3 months postoperatively (*P* = 0.018 and *P* = 0.010). No eyes in the irrigation group and five (3.9%) eyes in the nonirrigation group had macular edema (ME), highlighting a trend toward a greater incidence in the nonirrigation group (*P* = 0.06) (Figure 5).

## 4. Discussion

To our knowledge, our study is the first to use operating microscopy and iOCT to evaluate the intraoperative dynamics of the AC in real time. Microscopic evaluation is subjective, while iOCT evaluation is objective. The results showed a good agreement between the evaluation techniques for AC stability evaluation at five time points. Therefore, even without iOCT, ophthalmologists can evaluate AC stability using operating microscopy. The results of the iOCT evaluation revealed that the AC remained stable in only 4/123 eyes during phaco in the nonirrigation group.

The AC is prone to instability at T3 and T4; therefore, we designed an ACM. Agarwal [11] presented a trocar cannula as an ACM. Although the trocar ACM allows easy and atraumatic transconjunctival entry into the anterior segment and enables the creation of autosealing ports, surgeons who often perform bimanual maneuvers are not adept at using trocar ACM to assist with nucleus chopping. This increases the operative difficulty. Initially, we used a 26-gauge hydrodissection cannula as the irrigation tip of the ACM, which does not correctly match the size of the side incision, resulting in incision leakage, and it is easily blocked by nuclear fragments intraoperatively. Therefore, we chose a 22-gauge syringe needle as the irrigation tip of the ACM. Our ACM is easy to grasp and can replace the chopper to chop the nucleus during phaco. It enters the AC through a side incision without additional damage to the cornea. The irrigation tube used was a transfusion set with a valve that allowed free adjustment of the irrigation fluid. In our study, 102/121 eyes in the irrigation group had stable AC at five time points. Our ACM effectively maintained AC stability, particularly when the phaco and I/A tips were withdrawn from the eyes.

Some studies [12, 13] have suggested the breakdown of the blood-aqueous barrier (BAB), whose clinical features are flare and cells in the AC. Flare values and cell intensity peak on day 1 after phaco. Our data showed that the AC cell grades were significantly lower in the irrigation group at 1 day postoperatively. This indicates that stable AC during phaco may minimize surgery-induced inflammation. Maintaining intraoperative AC stability decreases the disturbance of the iris and incidence of iris prolapse, thus reducing the damage to the BAB.

To further investigate the effect of AC instability on intraocular tissues such as the cornea, vitreous, and retina, we followed up both groups postoperatively. Phaco inevitably results in loss of ECD, which is not renewable after damage [14]. Our study compared the postoperative ECD of patients in the two groups. We observed a postoperative reduction in ECD in both groups; however, the difference was not statistically significant. Several studies [15, 16] have reported that intraoperative fluctuation of AC increases corneal endothelial loss. Our ACM effectively maintains intraoperative AC stability, avoids AC shallowing and collapse, and provides adequate surgical space. Continuous AC irrigation also protects ECD from mechanical damage and thermal burns [15]. Milla et al. [6] compared postoperative ECD between groups based on whether an ACM was used. They found that the ACM maintained a constant anterior chamber volume during phaco without inducing any additional changes in the ECD.

**Figure 6:**
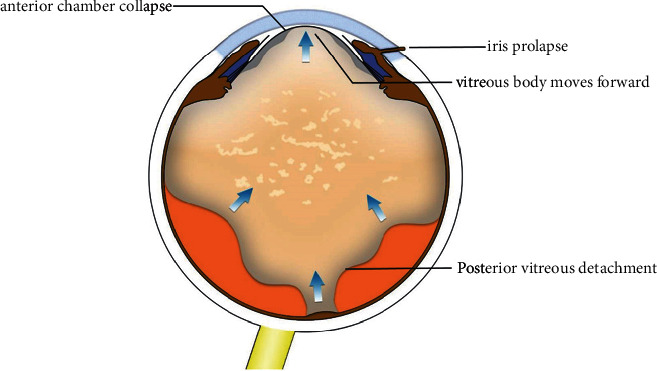
Schematic conceptual drawing of the anterior chamber collapse leading to vitreous anterior displacement.

Considering the eyeball as a whole, the posterior segment is also affected by the instability of the AC. Several studies [17–19] have found that phaco may cause or accelerate PVD. There are several hypothetical underlying mechanisms. First, the vitreous moves back and forth with repeated surging and collapse of the anterior and posterior chambers during cataract surgery, leading to traction of the posterior cortex (Figure 6). The traction may result in vitreous instability and weakening of vitreoretinal adhesion [17, 20]. Moreover, inflammation-induced degradation of hyaluronic acid and cross-linkage of vitreous collagen may promote the development of PVD [21, 22]. Our results revealed that 68.5% of eyes without preoperative PVD developed PVD at 3 months postoperatively in the nonirrigation group. Ivastinovicc et al. [18] followed up 49 patients who did not have PVD preoperatively for 3 months. In this study, some degree of PVD was observed in 71.4% of patients at 3 months postoperatively. In another study [17], PVD was detected in 70% of patients at 3 months. Our results are similar to those of previous studies. Only 28.6% of eyes without preoperative PVD developed PVD at 3 months postoperatively in the irrigation group. Maintaining AC stability during phaco significantly reduced the incidence of PVD postoperatively. We hypothesized that a stable AC weakens the traction on the posterior vitreous cortex, thus reducing the occurrence of PVD. Sudden AC collapse is a risk factor for destabilization of the vitreoretinal interface. The forward movement of the dynamic vitreous tract at the posterior border of the vitreous base increases the risk of retinal tear formation and even retinal detachment (RD) [23, 24]. Therefore, it is necessary to maintain AC stability to reduce the occurrence of PVD, retinal tears, and RD postoperatively.

Another finding was that anterior segment instability during phaco affected the retina. The CFT of the nonirrigation group was significantly higher than that of the irrigation group at 1 and 3 months postoperatively. Five (3.9%) eyes of five different patients had ME on OCT in the nonirrigation group. The incidence of ME after uncomplicated cataract surgery is multifactorial and appears to be related to postoperative inflammation induced by inflammatory mediators [25], which are released by the anterior uvea traversing the vitreous, reaching the posterior segment, and breaking down the blood-retinal barrier [26, 27]. We believe that effective maintenance of AC stability can reduce the incidence of ME for the two following reasons. First, stable AC can reduce the intraoperative disturbance in the iris, a metabolically active tissue that releases inflammatory mediators during trauma [25]. Second, mechanical stress plays a role in the pathogenesis of ME. Roldan et al. [28] have reported that ME is associated with incomplete PVD with vitreous traction. Traction of adhesions between the vitreous and macula results in irritation of Müller cells. [25]. Thus, AC stability is essential to minimize the risk of postoperative ME.

In our study, ACM effectively maintained AC stability, but AC instability was observed in 7.4% and 14.0% of eyes at T3 and T4. The main reason for this is that when chopping the nucleus using the irrigation tip, the nuclear fragments tend to block the tip, resulting in poor water flow. To avoid this, the assistant can squeeze the proximal ACM tube before the phaco tip is withdrawn from the AC to induce the discharge of nuclear fragments. Our approach was feasible and effective. There were also a few cases of AC instability due to excessive side incision resulting in fluid leakage. The size of the irrigation tip of the ACM must match the size of the side incision, and the 15º knife must be withdrawn along the tract of entry. Any lateral movement during entry or withdrawal produces a large incision and leakage. Surgeons should avoid excessive vertical and horizontal tension on the wound from surgical instruments while operating because such stress can cause wound deformation and leakage.

A relatively short follow-up duration is a limitation of our study. Moreover, maintaining AC stability is particularly important in high-risk eyes, such as those with high myopia, zonular laxity of the lens, or a history of retinal tears. Further studies including patients with high-risk eyes and with a longer follow-up period are needed to better assess the impact of AC instability on intraocular tissues.

## 5. Conclusions

In conclusion, the results of this study indicated that both operating microscopy and iOCT are efficient for AC stability evaluation. When the phaco and I/A tips were withdrawn from the AC, there was frequent instability. Intraoperative AC instability may increase surgery-induced inflammation and lead to postoperative complications such as PVD, RD, and ME. The self-made ACM can effectively reduce the incidence of intraoperative AC instability and the above-mentioned complications.

## Figures and Tables

**Table 1:** Demographic data and characteristics of enrolled patients.

	Irrigation group (eyes = 121)	Nonirrigation group (eyes = 127)	*P* value
Sex (male/female)	43/78	51/76	0.574
Age (years)	66.12 ± 7.73	65.36 ± 9.13	0.944
Lens grade (LOCS III)			
Cortical opacity	2.06 ± 0.96	2.19 ± 1.02	0.326
Nuclear opalescence	2.27 ± 0.46	2.29 ± 0.48	0.731
Posterior subcapsular opacity	2.35 ± 0.81	2.26 ± 0.80	0.366
AL (mm)	23.69 ± 1.74	23.82 ± 2.19	0.487
ACD (mm)	3.22 ± 0.42	3.17 ± 0.48	0.254

ACD: anterior chamber depth; AL: axial length; LOC: Lens Opacities Classification System.

**Table 2:** Incidence of AC instability at five time points in the two groups evaluated by operating microscopy and iOCT.

Time points	AC stability	Operating microscopy		*P* value	iOCT		*P* value
		Irrigation group (eyes = 121)	Nonirrigation group (eyes = 127)		Irrigation group (eyes = 121)	Nonirrigation group (eyes = 127)	
T1	Stability	119 (98.3)	124 (97.6)	0.691	117 (96.7)	122 (96.1)	0.790
	Instability	2 (1.7)	3 (2.4)		4 (3.3)	5 (3.9)	

T2	Stability	112 (92.6)	112 (88.2)	0.244	109 (90.1)	111 (87.4)	0.505
	Instability	9 (7.4)	15 (11.8)		12 (9.9)	16 (12.6)	

T3	Stability	113 (93.4)	34 (26.8)	<0.001 ^*∗*^	112 (92.6)	29 (22.8)	<0.001 ^*∗*^
	Instability	8 (6.6)	93 (73.2)		9 (7.4)	98 (77.2)	

T4	Stability	108 (89.3)	14 (11.0)	<0.001 ^*∗*^	104 (86.0)	9 (7.1)	<0.001 ^*∗*^
	Instability	13 (10.7)	113 (89.0)		17 (14.0)	118 (92.9)	

T5	Stability	106 (87.6)	110 (86.6)	0.816	108 (89.3)	107 (84.3)	0.246
	Instability	15 (12.4)	17 (13.4)		13 (10.7)	20 (15.7)	

AC: anterior chamber; iOCT: intraoperative optical coherence tomography.  ^*∗*^A significant *P* value.

**Table 3:** Consistency of operating microscopy and iOCT for observing AC stability in the irrigation group.

Time points	iOCT	Operating microscopy		Kappa	*P* value
		Stability	Instability		
T1	Stability	117	0	0.659	<0.001
	Instability	2	2		

T2	Stability	108	1	0.740	<0.001
	Instability	4	8		

T3	Stability	110	2	0.684	<0.001
	Instability	3	6		

T4	Stability	103	1	0.772	<0.001
	Instability	5	12		

T5	Stability	105	3	0.839	<0.001
	Instability	1	12		

AC: anterior chamber; iOCT: intraoperative optical coherence tomography.

**Table 4:** Consistency of operating microscopy and iOCT for observing AC stability in the nonirrigation group.

Time points	iOCT	Operating microscopy		Kappa	*P* value
		Stability	Instability		
T1	Stability	122	0	0.742	<0.001
	Instability	2	3		

T2	Stability	110	1	0.890	<0.001
	Instability	2	14		

T3	Stability	26	3	0.768	<0.001
	Instability	8	90		

T4	Stability	7	2	0.572	<0.001
	Instability	7	111		

T5	Stability	105	2	0.779	<0.001
	Instability	5	15		

AC: anterior chamber; iOCT: intraoperative optical coherence tomography.

**Table 5:** AC flare and cells grading 1 day postoperative with a clear cornea.

Grade	Irrigation group	Nonirrigation group	*P* value
	(eyes = 107)	(eyes = 110)	
AC flare			0.142
0	43	35	
1+	49	53	
2+	15	22	
AC cells			0.014 ^*∗*^
0.5+	27	17	
1+	55	53	
2+	19	27	
3+	6	13	

AC: anterior chamber. AC flare grade: 0 = none, 1 = faint, 2 = moderate, iris and lens detail clear; AC cells grade: 0.5 = 1–5 cells, 1 = 6–15 cells, 2 = 16–25 cells, 3 = 26–50 cells.  ^*∗*^ indicates a significant *P* value.

**Table 6:** Comparison of CDVA, IOP, ECD, and CCT.

Parameter	Irrigation group (eyes = 121)	*P* ^a^	Nonirrigation group (eyes = 127)	*P* ^a^	*P* ^b^
CDVA/logMAR					
Preop	0.48 ± 0.27	—	0.46 ± 0.26	—	0.610
1 week postop.	0.10 ± 0.12	<0.001 ^*∗*^	0.09 ± 0.10	<0.001 ^*∗*^	0.493
1 month postop.	0.05 ± 0.06	<0.001 ^*∗*^	0.06 ± 0.11	<0.001 ^*∗*^	0.157
3 months postop.	0.04 ± 0.07	<0.001 ^*∗*^	0.05 ± 0.08	<0.001 ^*∗*^	0.909
IOP/mmHg					
Preop.	14.87 ± 2.44	—	15.18 ± 2.45	—	0.295
1 week postop.	17.16 ± 5.49	<0.001 ^*∗*^	15.96 ± 3.78	0.044 ^*∗*^	0.092
1 month postop.	14.15 ± 2.12	0.056	13.79 ± 2.70	<0.001 ^*∗*^	0.245
3 months postop.	13.32 ± 2.54	<0.001 ^*∗*^	13.36 ± 2.52	<0.001 ^*∗*^	0.592
ECD (cell/mm^2^)					
Preop.	2491.09 ± 257.51	—	2509.15 ± 245.61	—	0.859
1 week postop.	2262.85 ± 232.42	<0.001 ^*∗*^	2248.46 ± 367.11	<0.001 ^*∗*^	0.755
1 month postop.	2274.65 ± 254.54	<0.001 ^*∗*^	2231.80 ± 314.64	<0.001 ^*∗*^	0.329
3 months postop.	2220.71 ± 271.00	<0.001 ^*∗*^	2192.73 ± 368.69	<0.001 ^*∗*^	0.567
CCT (*μ*m)		—			
Preop	528.44 ± 31.40	—	535.20 ± 30.89	—	0.170
1 week postop.	546.75 ± 35.22	<0.001 ^*∗*^	549.05 ± 41.14	<0.001 ^*∗*^	0.518
1 month postop.	536.37 ± 31.41	0.001 ^*∗*^	539.78 ± 35.17	0.006 ^*∗*^	0.520
3 months postop.	525.59 ± 29.86	0.188	529.80 ± 31.80	0.089	0.370

CDVA: corrected distance visual acuity; CCT: central corneal thickness; ECD: endothelial cell density; IOP: intraocular pressure; logMAR: logarithm of the minimum angle of resolution. P^a^: comparison between preop. and postop.; P^b^: comparison of both groups.  ^*∗*^ indicates a significant *P* value.

## Data Availability

The data used to support the findings of this study are available from the corresponding author upon request.
